# Preliminary volume−based optimization of radioactive iodine dose without radioactive iodine uptake in Graves’ disease

**DOI:** 10.3389/fendo.2026.1823030

**Published:** 2026-05-14

**Authors:** Keattichai Keeratitanont, Sornsupha Limchareon, Sutasinee Kongpromsuk, Watanya Jaidee, Teetawat Jearsirikul, Chantham Sunmahakhun

**Affiliations:** Department of Radiology and Nuclear Medicine, Burapha University, Chonburi, Thailand

**Keywords:** dose–response relationship, Graves’ disease, radioactive iodine therapy, thyroid volume, treatment-related adverse events

## Abstract

**Background:**

Optimal radioactive iodine (RAI) dosing for Graves’ disease remains controversial. Conventional calculated regimens require radioactive iodine uptake (RAIU) testing, whereas empiric fixed-dose strategies risk under- or overtreatment. Although activity per gram of thyroid tissue provides an uptake-independent approach, volume-specific minimum effective doses and functional outcomes remain insufficiently defined. This study aimed to determine the minimum RAI activity per gram required to achieve ≥75% remission across stratified thyroid volumes, to identify the activity associated with the highest euthyroid remission, and to evaluate whether dose escalation increases treatment-related adverse events.

**Methods:**

This retrospective study included 236 patients with Graves’ disease who received initial single-dose RAI therapy between August 2022 and August 2024 at a tertiary center in Thailand. Thyroid volume by ultrasonography was categorized as <25 g, 25–49.9 g, 50–74.9 g, and ≥75 g. Administered activity was grouped as <0.30, 0.30–0.39, 0.40–0.49, 0.50–0.59, and ≥0.60 mCi/g. Outcomes and adverse events were assessed at 6 months.

**Result:**

The minimum activity required to achieve ≥75% remission increased with thyroid volume: 0.30–0.39 mCi/g for <25 g glands (90%), 0.40–0.49 mCi/g for 25–49.9 g (77%), and 0.50–0.59 mCi/g for 50–74.9 g (100%). No activity achieved ≥75% remission in glands ≥75 g, with a maximum rate of 45%. Across all volumes, the highest euthyroid rates occurred at the lowest effective activity, whereas dose escalation increased hypothyroidism without improving overall remission. Adverse events were infrequent (5.5–7.6%) and mostly mild. Moderate neck pain was more frequent at higher activities, with a strong positive correlation between administered activity and total adverse events (r = 0.82, p = 0.089).

**Conclusion:**

This study demonstrates that volume−stratified radioiodine dosing offers an effective and practical alternative to traditional 24−hour RAIU–based dosimetry for Graves’ disease. Adjusting activity according to thyroid volume yields predictable remission while avoiding the logistical burdens of uptake testing. The dose–response relationship also underscores the need to balance efficacy with avoidance of excess toxicity. Overall, this individualized, volume−based strategy provides a scalable and efficient framework for optimizing radioiodine therapy, particularly in resource−limited settings.

## Introduction

Graves’ disease is the most common cause of hyperthyroidism worldwide and remains a significant contributor to cardiovascular morbidity, metabolic dysfunction, and impaired quality of life. Definitive management strategies include antithyroid drugs, thyroidectomy, and radioactive iodine (RAI) therapy (1), with RAI representing a widely utilized, cost-effective, and durable treatment option in many regions (1, 2). Despite decades of clinical use, the optimal method for determining RAI activity remains controversial.

Traditional calculated dosing protocols incorporate 24-hour radioactive iodine uptake (RAIU) testing and thyroid gland size estimation to individualize therapy, as recommended in major practice guidelines (1, 3). However, RAIU-based dose estimation is associated with logistical complexity, inter-institutional variability, and inconsistent remission rates (4–6). In contrast, empiric fixed-dose strategies (commonly 10–15 mCi) offer operational simplicity but frequently demonstrate heterogeneous outcomes and may either undertreat large glands or overtreat small glands, thereby increasing the risk of permanent hypothyroidism and other radiation-related adverse events (7–10).

Emerging evidence suggests that RAI activity calculated per gram of thyroid tissue provides a physiologically grounded dosing strategy that can be implemented without the use of RAIU testing. Recent retrospective studies have identified universal thresholds (e.g., ≥0.4 mCi/g) associated with remission rates exceeding 85% (11). However, applying a single activity-per-gram cutoff across all patients may inadequately account for heterogeneity in baseline thyroid volume, particularly in individuals with marked gland enlargement in whom reduced therapeutic responsiveness has been observed (12, 13). Furthermore, although remission is typically defined biochemically, the functional outcome of euthyroidism versus iatrogenic hypothyroidism remains clinically consequential, as excessive administered activity may increase the risk of permanent hypothyroidism and lifelong thyroid hormone replacement. Evidence defining the minimum effective RAI activity across stratified thyroid volume categories remains limited, and it is unclear whether dose escalation beyond therapeutic thresholds improves euthyroid remission or instead disproportionately increases treatment-related adverse events.

Accordingly, this study seeks to refine RAI dosing protocols to optimize the therapeutic index of definitive treatment for Graves’ disease, specifically by bypassing the requirement for RAIU measurements. The primary objective is to establish the minimum RAI activity per gram of thyroid tissue necessary to maintain a remission threshold exceeding 75% across stratified thyroid volume categories. The secondary objective aims to identify the optimal dose that maximizes euthyroid outcomes while mitigating the risk of iatrogenic hypothyroidism. Finally, the study evaluates the dose-response relationship between administered RAI activity and the incidence of treatment-related adverse events.

## Materials and methods

### Patient selection

This retrospective cohort study evaluated the clinical records of patients diagnosed with Graves’ disease who received their initial single-dose radioactive iodine (RAI) therapy at Burapha University Hospital, Thailand, between August 2022 and August 2024. The comprehensive dataset extracted from the hospital’s electronic health records system included baseline demographics, thyroid gland volume, administered RAI activity, adverse event profiles, and post-treatment remission rates. Patients were excluded from the study if they had incomplete clinical data, a history of previous RAI therapy or thyroidectomy, documented non-compliance, or an inability to safely discontinue medications known to interfere with RAI uptake (1, 3), concurrent thyroid cancer, prior thyroid surgery before RAI treatment evaluation, and adjunctive treatments other than antithyroid medication, such as herbal medicine or percutaneous ethanol injection. The study was approved by the Burapha University Ethics Committee (identifier number: HS099/2567).

### Thyroid size assessment

Thyroid gland dimensions were obtained from neck ultrasonography images and interpreted by a board-certified diagnostic radiologist. Thyroid volume was calculated using the standard ellipsoid formula (volume = depth × length × width × π/6), as previously validated by Chanoine et al. (1991), from which the estimated thyroid weight was derived (14). For analytical purposes, thyroid sizes were stratified into four categories: < 25 g, 25 to 50 g, 50 to 75 g, and ≥ 75 g.

### Patient preparation and treatment

To optimize radiotracer uptake, all patients were instructed to discontinue antithyroid drugs (methimazole or propylthiouracil) for 3 to 5 days prior to RAI administration. Potassium iodide was not administered to any patient in this cohort. In patients with prior exposure to high-iodine substances, including Lugol’s solution or iodinated contrast media, RAI administration was postponed for a minimum of 4 weeks. Additionally, all patients were instructed to adhere to a strict low-iodine diet for at least one week prior to RAI administration, which included avoidance of high-iodine foods such as seafood, fish sauce, and iodized salt. Non-iodized salt was provided by the department to all patients scheduled for RAI therapy to facilitate dietary compliance. Patients with suspected thyroid-associated ophthalmopathy underwent a comprehensive evaluation by an ophthalmologist before clearance for treatment, and routine pregnancy testing was performed on all premenopausal female patients on the day of therapy.

Administered RAI activities were determined based primarily on estimated thyroid weight and clinical urgency, without 24-hour RAIU testing. Higher activity-per-gram ratios were preferentially selected in patients with urgent indications, including thyroid storm, thyrotoxic periodic paralysis, or significant cardiovascular disease. The institutional regulatory limit of 30 mCi constrained the achievable dose-per-gram ratio in patients with markedly enlarged glands. To evaluate the dose-response relationship, the administered activity per gram of thyroid tissue was calculated and categorized as < 0.30, 0.30–0.39, 0.40–0.49, 0.50–0.59, and ≥ 0.60 mCi/g. Following administration, all patients were mandated to strictly adhere to standard radiation safety protocols for a minimum of one week.

### Outcome measurement

Clinical and biochemical treatment outcomes were systematically evaluated at 6 months post-RAI administration via in-person clinic visits or, for those unable to attend, telephone follow-up. Treatment efficacy was divided into persistent disease or remission. Persistent disease was defined biochemically by suppressed thyroid-stimulating hormone (TSH) levels with normal or elevated serum free thyroxine (FT4) and free triiodothyronine (FT3), and clinically by the ongoing requirement for antithyroid drugs (ATDs). Remission was defined as the achievement of either a euthyroid or hypothyroid state. Euthyroidism was characterized by normalized TSH, FT4, and FT3 levels without the requirement for ATDs or thyroid hormone replacement therapy, whereas hypothyroidism was diagnosed upon the presentation of low serum FT4 and/or FT3 levels alongside elevated TSH levels, or if the patient had actively been initiated on thyroid hormone replacement. For dose-optimization analyses, a remission rate of ≥75% was predefined as the institutional benchmark for clinically acceptable therapeutic efficacy. Within each thyroid volume category, the lowest administered activity achieving this threshold was defined as the minimum effective dose.

### Adverse events

Adverse events, excluding anticipated therapy-induced hypothyroidism, were systematically documented and stratified into three severity grades. Mild events were defined as minimal, self-limiting symptoms resolving spontaneously within 1 to 2 days without pharmacological intervention. Moderate events required short-term medical therapy (3 to 5 days) or a single outpatient hospital evaluation, while severe events necessitated long-term pharmacological management, continuous medical supervision, or hospital admission.

### Statistical analysis

Baseline demographic and clinical characteristics of this study were summarized using standard descriptive statistics. Continuous variables, including patient age, medication dosage, treatment duration, thyroid gland volume, and administered radioactive iodine activity per gram of the thyroid gland, were evaluated for normal distribution and are expressed as means with standard deviations. Categorical variables, such as patient sex, primary indications for treatment, clinical outcomes, and the incidence of adverse events, are presented as absolute frequencies and percentages. To evaluate therapeutic efficacy, patients were stratified into subgroups based on baseline thyroid volume and the administered activity per gram of tissue. Differences in categorical outcomes, specifically comparing the rates of remission, euthyroidism, and adverse events across the various dosing groups, were analyzed using Pearson’s chi square test or Fisher’s exact test. Spearman’s rank correlation analysis was employed to assess the relationship between RAI dosage and the percentage of treatment-related adverse events. For all comparative analyses, a two-sided p value of less than 0.05 was considered statistically significant using SPSS software version 28.0.

## Results

A total of 268 patients with hyperthyroidism who visited the outpatient department of nuclear medicine between August 2022 and August 2024 were initially assessed. After applying exclusion criteria, 12 were lost to follow-up or lost contact, 6 had insufficient data, 5 were unable to discontinue medications affecting RAI treatment, 5 had undergone prior thyroid surgery, 3 had previously been treated with RAI, and 1 had thyroid cancer.

The remaining 236 patients were eligible for the study. The mean age of the patients was 39 years and 2 months, comprising 160 (67.8%) females and 76 (32.2%) males. Methimazole (MMI) was prescribed to 211 patients (89.4%) at a mean dosage of 13.6 ± 8.1 mg per day, and propylthiouracil (PTU) was prescribed to 25 patients (10.6%) at a mean dosage of 483.0 ± 303.0 mg per day. The average duration of antithyroid drug treatment (including MMI and PTU) was 4 years and 5 months. The mean thyroid gland size was 36.7 ± 27.1 g. The administered RAI activity ranged from 0.16 to 1.06 mCi per gram of thyroid tissue, with a mean activity of 0.42 mCi per gram. The mean patient follow-up time was 6 months and 15 days. The primary indications for RAI treatment were medication failure (109 patients), relapsed disease (46 patients), thyrotoxicosis periodic paralysis (27 patients), thyroid storm (26 patients), Graves’ ophthalmopathy (5 patients), and other conditions such as heart disease (27 patients), stroke (5 patients), antithyroid drug side effects (6 patients), and miscellaneous reasons (2 patients) (**Table 1**).

**Table 1:** Baseline characteristic (N = 236).

Characteristic	Values
Age (years:month)	
Mean (SD)	39:2 (12:5)
Range	15 – 82
Gender, n (%)	
Female	160 (67.5)
Male	77 (32.5)
Time before RAI treatment (years:month)	
Mean (SD)	4:5 (4:1)
Range	0:1 - 26:0
Thyroid gland size (gram)	
Mean (SD)	36.9 (27.2)
Range (g)	8.0 - 183.9
RAI activity (mCi/gram)	
Mean (SD)	0.42 (0.14)
Range	0.16 – 1.06
Follow-up time (months)	
Mean (SD)	6:12 (0:23)
Range	5:15 - 10:7
Indication for RAI treatment	
Medication failure	109
Relapsed disease	46
Thyrotoxicosis periodic paralysis	27
Thyroid storm	26
Antithyroid drug side effects	6
Graves’ ophthalmopathy	5
Heart disease^*^	27
Stroke	5
Miscellaneous^#^	2

^*^ Heart disease encompassed atrial fibrillation (AF), Wolff–Parkinson–White syndrome (WPW), bigeminy premature ventricular complexes, valvular heart disease, and single-vessel coronary artery disease.

^#^ Miscellaneous indications included renal infarction and cirrhosis.

### Optimal radioactive iodine activity for remission

**Figure 1:**
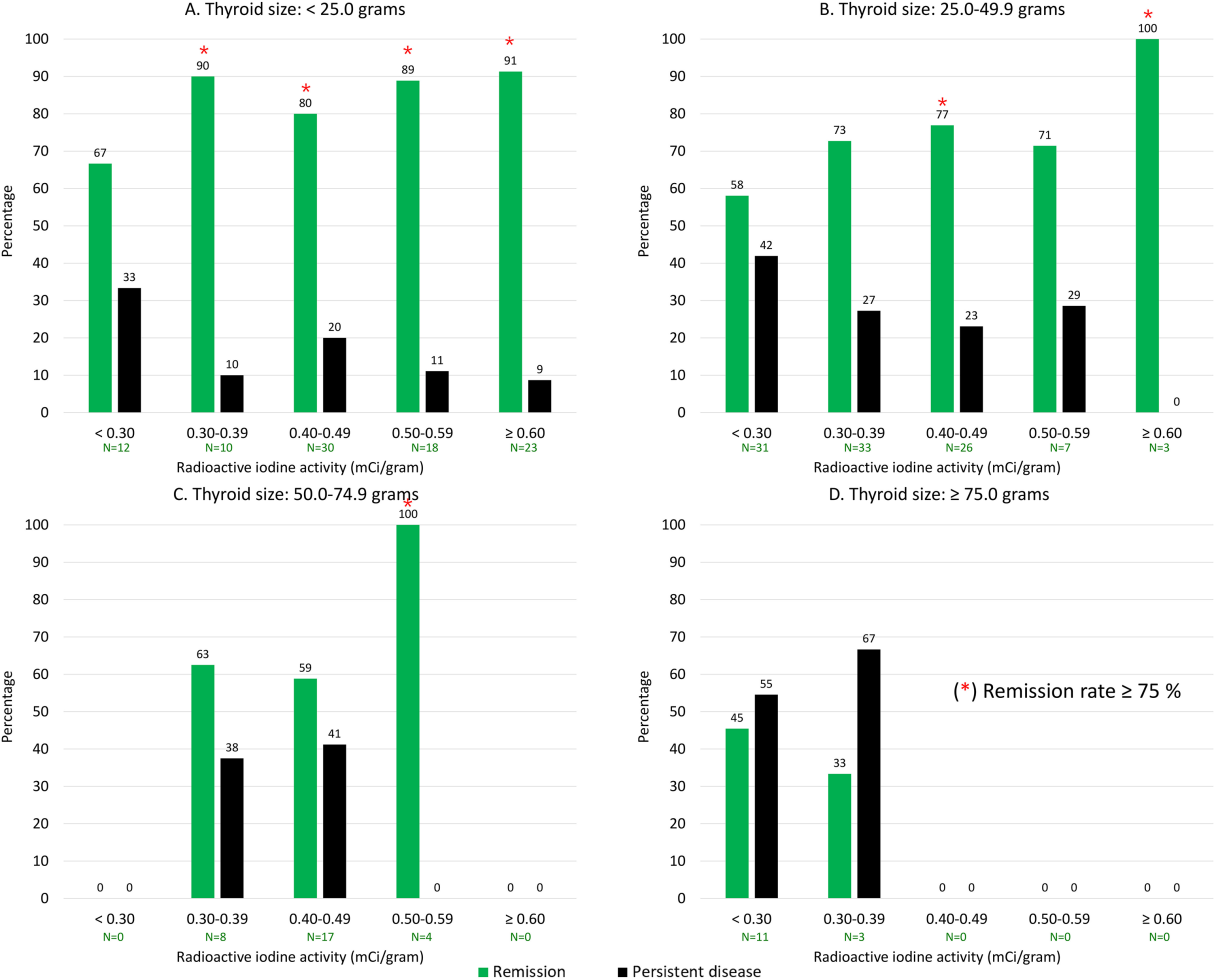
Remission rates following radioactive iodine (RAI) therapy stratified by administered activity (mCi/g) and thyroid size categories: **(A)** < 25.0 grams, **(B)** 25.0–49.9 grams, **(C)** 50.0–74.9 grams, and **(D)** ≥ 75.0 grams.

The minimum radioactive iodine (RAI) activity required to achieve a ≥75% remission rate scaled with baseline thyroid volume. The number of patients in each thyroid volume and activity subgroup is summarized in **Supplementary Table 1**, and the corresponding distribution of patients achieving remission is presented in **Supplementary Table 2**. In small glands (<25.0 g), this threshold was consistently met at all activities >0.3 mCi/g, beginning at the minimum effective dose of 0.30–0.39 mCi/g (90%, 9 of 10) and remaining stable across higher dosing tiers up to ≥0.6 mCi/g (91%, 21 of 23). Intermediate volumes (25.0–49.9 g) required higher administered activities to reach therapeutic efficacy, with the ≥75% target first achieved at 0.40–0.49 mCi/g (77%, 20 of 26) and peaking at ≥0.6 mCi/g (100%, 3 of 3). In glands measuring 50.0–74.9 g, the minimum effective activity further escalated to 0.50–0.59 mCi/g (100%, 4 of 4). Notably, therapeutic efficacy was blunted in patients with marked thyromegaly (≥75 g), as no evaluated RAI activity reached the ≥75% threshold; the maximum observed remission in this cohort was 45% (5 of 11) at <0.3 mCi/g (**Figure 1**).

### Optimal radioactive iodine activity for euthyroid remission

**Figure 2:**
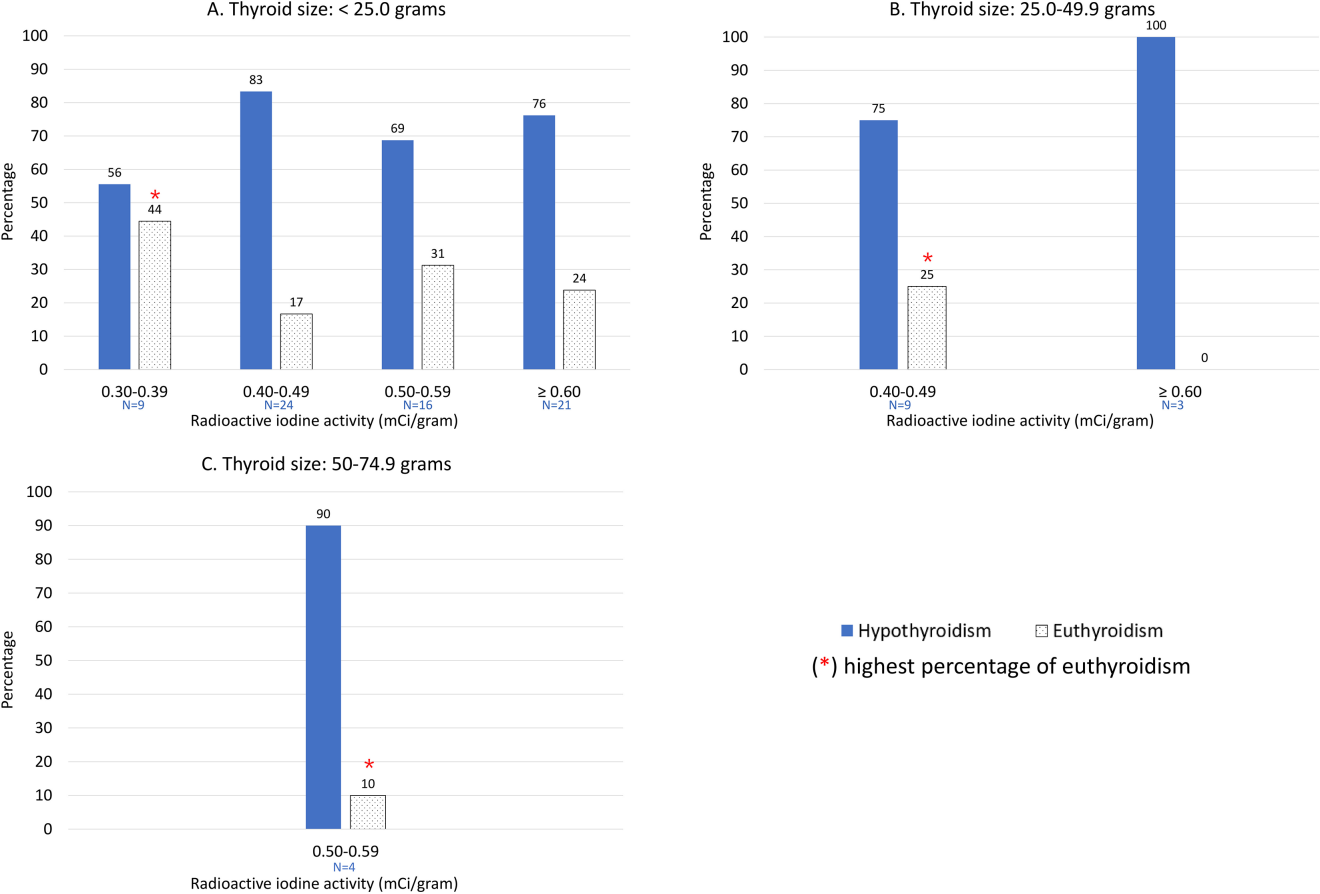
Post-treatment thyroid functional outcomes (hypothyroidism and euthyroidism) according to administered RAI activity within each thyroid size category: **(A)** < 25.0 grams, **(B)** 25.0–49.9 grams, and **(C)** 50–74.9 grams.

In a secondary analysis of patients meeting the ≥75% remission threshold, functional outcomes varied according to the administered radioactive iodine (RAI) activity. Across all evaluated gland volumes, the highest proportion of patients maintaining euthyroidism was observed at the lowest effective RAI dose. In small glands (<25.0 g), euthyroidism was most frequent (44%, 4 of 9) at the minimal effective activity of 0.30–0.39 mCi/g. Dose escalation was associated with an increased incidence of hypothyroidism and a corresponding decline in euthyroid rates to 17% (4 of 24) at 0.40–0.49 mCi/g, 31% (5 of 16) at 0.50–0.59 mCi/g, and 24% (5 of 21) at ≥0.60 mCi/g. Similarly, in intermediate volume (25.0–49.9 g), the lowest effective dose (0.40–0.49 mCi/g) yielded euthyroidism in 25% (5 of 20) of patients, whereas escalation to ≥0.60 mCi/g resulted in hypothyroidism in all patients (0% euthyroid). In large glands (50.0–74.9 g), the sole effective activity level (0.50–0.59 mCi/g) resulted in hypothyroidism in 90% (9 of 10) of patients, with 10% (1 of 10) remaining euthyroid (**Figure 2**).

### Safety and adverse events

Treatment was well tolerated across all dosing multipliers, with a consistently low incidence of adverse events across groups. The overall rate of total adverse events ranged narrowly from 5.5% to 7.6%, without marked variation between dosing categories. Most reported events were mild in severity, occurring in 5.5%, 5.7%, 4.1%, 3.3%, and 3.8% of patients receiving <0.30, 0.30–0.39, 0.40–0.49, 0.50–0.59, and ≥0.60 mCi/g, respectively. Moderate adverse events were infrequent and observed only at doses ≥0.40 mCi/g. Moderate adverse events were rare and occurred only at doses ≥0.40 mCi/g, with one case reported in each of the 0.40–0.49 (1.4%), 0.50–0.59 (3.3%), and ≥0.60 (3.8%) groups, while none were observed at <0.40 mCi/g. No severe adverse events were documented in any dosing category. The distribution of adverse event severity and specific event types according to dosing multiplier is summarized in **Table 2**.

**Table 2:** Adverse events following RAI therapy in Graves’ disease.

Adverse event	Radioactive iodine activity (mCi/g)				
	< 0.30	0.30-0.39	0.40-0.49	0.50-0.59	≥ 0.60
Mild, N (%)	2 (3.7)	3 (5.7)	3 (4.1)	1 (3.3)	1 (3.8)
Neck pain	1	2	1	0	1
Dry mouth	1	0	2	0	0
Headache	1	0	0	0	0
Exacerbate thyrotoxicosis	0	1	0	0	0
Neck swelling	0	0	0	1	0
Moderate, N (%)	0 (0)	0 (0)	1 (1.4)	1 (3.3)	1 (3.8)
Neck pain	0	0	1	1	1

### The relationship between RAI activity and the percentage of adverse events.

**Figure 3:**
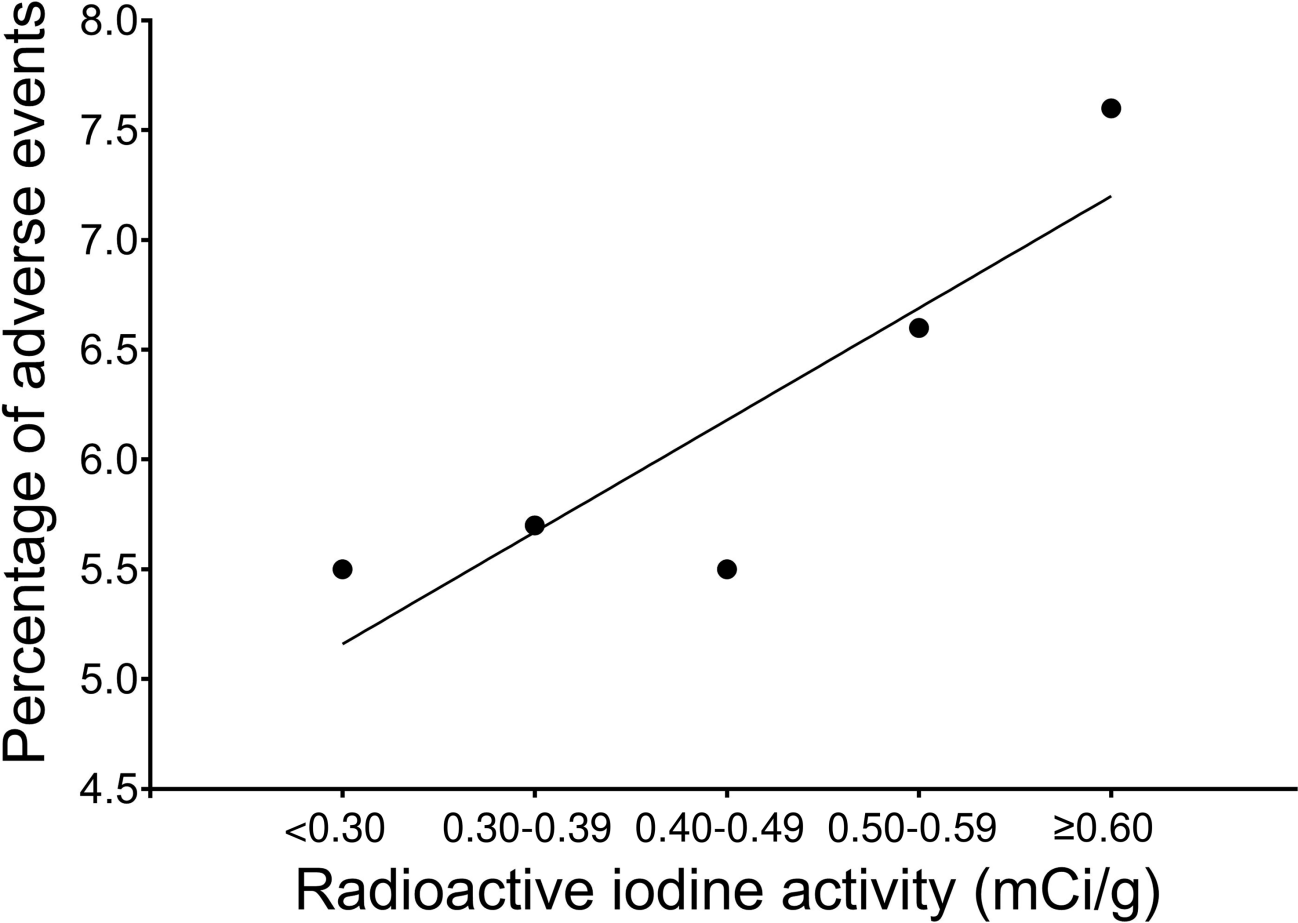
Relationship between administered RAI activity (mCi/g) and overall adverse event rate.

The relationship between the administered dose of RAI and the incidence of treatment-related adverse events was evaluated using Spearman’s rank-order correlation. Although a strong positive association was observed (r = 0.82), the correlation did not reach statistical significance (p = 0.089, N = 5). These findings suggest a notable clinical trend wherein higher RAI dosages correlate with increased adverse event rates (**Figure 3**).

## Discussion

This study demonstrates that a strict volume-stratified threshold for calculating RAI activity achieves highly predictable remission rates without the need for traditional 24-hour RAIU testing. By indexing the minimum effective activity to baseline thyroid volume, we have established a granular dosing hierarchy: 0.30–0.39 mCi/g is sufficient for small glands <25.0 g, yielding >90% remission, whereas intermediate (25.0–49.9 g) and large (50.0–74.9 g) glands require escalating activities of 0.40–0.49 mCi/g and 0.50–0.59 mCi/g, respectively, to reliably exceed a 75% remission threshold. Parallel to this efficacy-driven escalation, our correlation analysis demonstrated a strong positive association between administered RAI dose and the incidence of treatment-related adverse events (r = 0.82), although this did not reach conventional statistical significance (p = 0.089).

Our findings provide a critical refinement to the evolving body of literature advocating for the omission of routine 24-hour RAIU studies. Recent evidence, notably the 2024 analysis by Mihailescu et al., demonstrated that RAI can be effectively administered without prior uptake testing, identifying a universal threshold of 0.4 mCi/g as an independent predictor of therapeutic success (85.9% remission) (11). Similarly, personalized dosing concepts independent of thyroid uptake have yielded remission rates exceeding 86% (15). While our data support the fundamental premise of these methodologies, our volume-stratified outcomes suggest that a universal target may lack the granular precision required for heterogeneous thyroid volumes. By implementing a volume-dependent sliding scale, our approach prevents unnecessary radiation exposure in patients with small glands—who achieve excellent remission at >0.3 mCi/g—while simultaneously mitigating the systemic undertreatment of larger glands (50.0–74.9 g), which our data indicate strictly require ≥ 0.5 mCi/g to ensure therapeutic efficacy.

Furthermore, this study challenges the sustained clinical utility of conventional calculated dosing. Traditional dosimetry relies on formulas that mandate a 24-hour RAIU assessment, yet the literature evaluating this approach consistently reports highly variable remission rates ranging from 60% to 90% (5, 13, 16–18), This variability is likely driven by fluctuating uptake dynamics, detector calibration inconsistencies, and heterogeneous institutional protocols. Bypassing the RAIU test not only eliminates these physiological and technical variables but also significantly reduces logistical burdens and healthcare costs. Consequently, our volume-stratified model is highly translatable to resource-limited settings, offering a streamlined workflow without compromising curative outcomes (4–6). In Thailand, as in many middle-income countries, nuclear medicine infrastructure remains concentrated in metropolitan tertiary centers, with only 21 public facilities serving 75 provinces across all regional areas of the country. This means most provinces lack a dedicated nuclear medicine facility, requiring patients to travel across provincial boundaries for RAI therapy (19). Under a conventional RAIU-based protocol, this journey must be made twice, once for uptake measurement and again for treatment, imposing substantial time and financial burdens on patients. Eliminating the RAIU step through a volume-stratified approach consolidates care into a single visit, reducing costs and improving accessibility. This framework is therefore broadly applicable across Southeast Asia and other regions where nuclear medicine resources remain limited (20).

Importantly, our data highlight the inherent physiological limitations of fixed-dose empiric methods (typically 10–15 mCi). Despite their logistical convenience, fixed doses often correlate with suboptimal remission rates compared to calculated strategies (8–10). Although escalating fixed doses to 20 mCi can improve outcomes, multiple studies demonstrate a dose-response plateau where further activity confers diminishing returns but disproportionate toxicity (7–9), multiple studies demonstrate a dose-response plateau where further activity confers minimal benefit but disproportionate toxicity (21). Our data contextualize this plateau: because outpatient regulatory limits often cap administration at approximately 30 mCi, patients with massive glands (>75 g) inherently receive an inadequate dose-per-gram ratio. Our model thus delineates the upper threshold of RAI efficacy; patients with marked thyroid enlargement exhibited blunted responses with a maximum remission rate of only 45%, consistent with recent observations (12, 13). For such cases, alternative definitive interventions, such as thyroidectomy or fractionated strategies, should be prioritized. In patients who did not achieve remission after the first RAI treatment, subsequent therapy was highly effective. In our cohort, 59 of 63 patients achieved hypothyroidism after the second RAI treatment. Three patients were maintained on low-dose methimazole, and one required a third course of RAI. These findings support the role of repeated RAI as an effective strategy in initial non-responders.

Ultimately, this study suggests that calibrating the minimum therapeutic activity to specific gland volumes optimizes the risk-benefit ratio. Unnecessary escalation of activity exacerbates iatrogenic toxicity, including radiation thyroiditis (9, 22), exacerbation of Graves’ ophthalmopathy (23), and unintended salivary gland injury (24). In this study, administered activity demonstrated a strong positive correlation with adverse events (r = 0.82), although this did not reach conventional statistical significance (p = 0.089), likely reflecting limited statistical power. Nevertheless, the magnitude and direction of the association suggest a clinically meaningful dose–toxicity relationship and raise the possibility of a threshold beyond which further dose escalation disproportionately increases morbidity. These findings reinforce the importance of individualized, volume-based dosing to preserve the therapeutic window while minimizing dose-dependent adverse outcomes.

Limitations

Several limitations warrant consideration. First, the retrospective design inherently relied on historical clinical data and limited control over potential unmeasured confounders. Second, thyroid volume estimation by ultrasound is operator-dependent, and inter- and intra-observer variability were not formally assessed in this study. Third, although overall cohort size was substantial, stratification by baseline thyroid volume and administered activity resulted in small sample sizes within the largest gland volume tiers, thereby limiting statistical power in patients with markedly enlarged thyroid glands. Finally, systemic symptoms such as palpitations, diarrhea, or fever were not included in the routine adverse event assessment checklist, as these events are rare following RAI therapy in Graves’ disease, and future prospective studies should incorporate a more comprehensive symptom assessment.

## Conclusion

This study supports a volume-stratified radioiodine dosing strategy without reliance on RAIU as an effective and pragmatic alternative to conventional 24-hour RAIU-based dose estimation in Graves’ disease. Tailoring administered activity to thyroid volume yielded predictable remission across defined gland size categories while obviating the logistical and economic constraints of RAIU testing. The observed dose–response pattern, including a positive association between higher administered activity and adverse events, underscores the importance of preserving the therapeutic window. Although larger glands required higher activity to achieve remission, careful calibration appears necessary to minimize dose-related toxicity. Collectively, these findings suggest that an individualized, volume-based dosing framework may enhance the precision, feasibility, and scalability of radioiodine therapy, particularly in resource-constrained settings.

## Data Availability

The raw data supporting the conclusions of this article will be made available by the authors, without undue reservation.
