# Supplementary material for: Preliminary volume−based optimization of radioactive iodine dose without radioactive iodine uptake in Graves’ disease
Source: Front Endocrinol (Lausanne). 2026 May 14;17:1823030. doi: 10.3389/fendo.2026.1823030 (PMC13215940; doi:10.3389/fendo.2026.1823030)
Supplement: Supplementary file 1 [file Table1.docx]

| **Table S1**: Distribution of patients by thyroid volume category and administered radioactive iodine activity per gram of thyroid tissue (N = 236) | | | | | |
| --- | --- | --- | --- | --- | --- |
| **Thyroid size** | **Radioactive iodine activity (mCi/g)** | | | | |
|  | < 0.30 | 0.30-0.39 | 0.40-0.49 | 0.50-0.59 | ≥ 0.60 |
| < 25.0 | 12 | 10 | 30 | 18 | 23 |
| 25.0-49.9 | 31 | 33 | 26 | 7 | 3 |
| 50.0-74.9 | 0 | 8 | 17 | 4 | 0 |
| ≥75 | 11 | 3 | 0 | 0 | 0 |

| **Table S2**: Distribution of patients achieving remission by thyroid volume category and administered radioactive iodine activity per gram of thyroid tissue (N = 173) | | | | | |
| --- | --- | --- | --- | --- | --- |
| **Thyroid size** | **Radioactive iodine activity (mCi/g)** | | | | |
|  | < 0.30 | 0.30-0.39 | 0.40-0.49 | 0.50-0.59 | ≥ 0.60 |
| < 25.0 | 8 | 9 | 24 | 16 | 21 |
| 25.0-49.9 | 18 | 24 | 20 | 5 | 3 |
| 50.0-74.9 | 0 | 5 | 10 | 4 | 0 |
| ≥75 | 5 | 1 | 0 | 0 | 0 |
